# A Unique Set of Symptoms in the Background of SARS-CoV-2 Infection

**DOI:** 10.7759/cureus.14244

**Published:** 2021-04-01

**Authors:** Muhammad Ammar B Hamid, Shahan Tariq

**Affiliations:** 1 Internal Medicine, National University of Medical Sciences, Rawalpindi, PAK

**Keywords:** severe acute respiratory syndrome coronavirus 2, covid-19, sars-cov-2

## Abstract

Coronavirus disease 2019 (COVID-19) is a respiratory disease that has been reported to have a wide array of extrapulmonary manifestations. Our knowledge regarding this virus is constantly evolving, and new literature is being published every day. Children usually have a mild severity of COVID-19 infection. A variety of skin lesions have been documented in this disease. Similarly, our 18-month-old patient who was diagnosed with severe acute respiratory syndrome coronavirus 2 (SARS-CoV-2) infection had mild symptoms, but three days later he presented to the hospital with the development of urticarial lesions followed by angioedema. In this case report, we have attempted to highlight a possible association of angioedema and urticarial with coronavirus infection. Physicians should be aware of this association and should always inquire about symptoms of respiratory illness (SARS-CoV-2) while dealing with patients in whom a specific trigger for angioedema/urticaria is un-identifiable.

## Introduction

The severe acute respiratory syndrome coronavirus 2 (SARS-CoV-2) infection has spread globally on a massive scale. It continues to pose a major challenge to the healthcare community. The pediatric population represents only a small proportion (5%) of the total number of coronavirus disease 2019 (COVID-19) patients [1]. COVID-19 has been linked to a variety of different dermatological conditions. Some of the documented manifestations in children include maculopapular rash, erythema multiforme, vesicular rash, chilblain-like lesions, multisystem inflammatory syndrome, and urticaria [2]. The combination of both urticaria and angioedema in a COVID-19 patient has rarely been reported in the literature. The purpose of this case report is to enlighten healthcare professionals regarding the possible development of angioedema in a SARS-CoV-2 patient. Here we describe a case that presented to us with urticaria and subsequently angioedema in the background of SARS-CoV-2 infection.

## Case presentation

An 18-month-old boy presented to the hospital with a five-day history of fever and cough. The patient was in his usual state of health when he started to develop a fever. It was gradual in onset and continuous in character. It was documented by the mother using a clinical thermometer and was mostly low-grade in severity. The fever was relieved by the use of paracetamol syrup. Rigors or chills were not reported. In addition, the patient also complained of a dry cough; however, there was no history of vomiting. It started around the same time as the onset of fever and was of mild severity continually occurring throughout the day. It had no specific aggravating or relieving factors. He had been eating and drinking normally since the onset of symptoms. On further inquiry, there was no history of shortness of breath, weight loss, sleep disturbance, or overall patient distress. His past medical history was unremarkable, and there was no recent travel history. No specific allergies were reported. He was breastfed up till the age of one year and any formula feed was not used. He was up to date on his vaccinations and had achieved all the developmental milestones at the appropriate respective age. The parents did not report any similar symptoms over the past week. He did not have any siblings.

On examination, he had a low-grade fever of 100°F. The rest of the vital signs were normal. Mucous membranes were moist, and there was no evidence of pallor, jaundice, or cyanosis. Inspection of the throat revealed pharyngeal erythema; however, tonsils were not enlarged. The respiratory examination revealed normal chest rise and bilateral vesicular breath sounds. No rhonchi or rales could be appreciated. Cardiovascular and abdominal examinations were normal. Baselines along with a rapid streptococcal antigen test were negative. The patient was advised to undergo COVID-19 PCR (polymerase chain reaction) test, and the parents were recommended to take precautionary measures. On the next day, the patient’s COVID-19 PCR came back positive. The parents were counseled to wear facemasks, practice hand hygiene, and continue conservative management.

**Figure 1:**
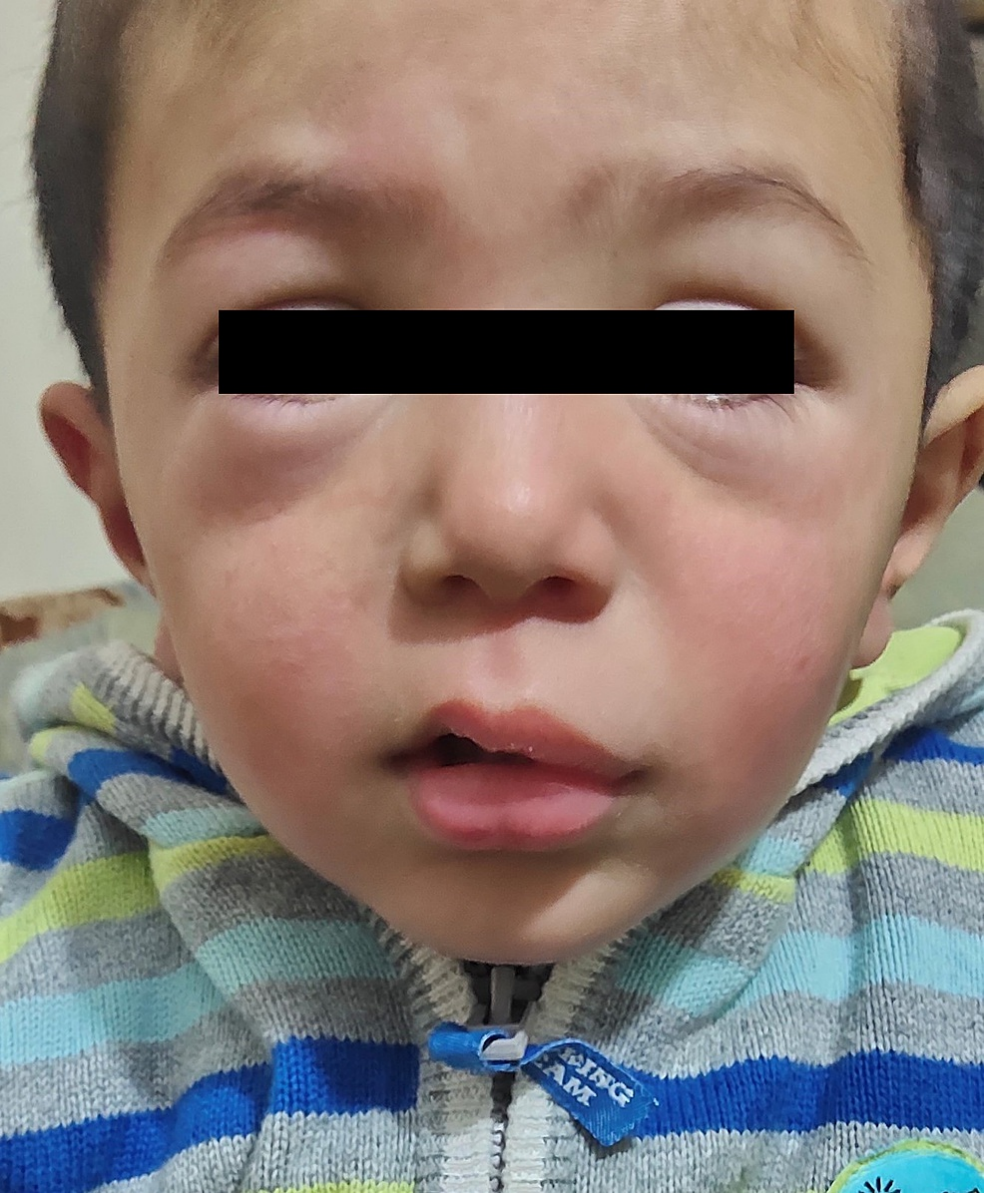
Swelling around the eyes and lips representing angioedema

After three days, the child was brought to the hospital with complaints of a generalized itchy skin rash followed by the development of swelling around the eyes and lips (Figure 1). There was no history of atopy, insect bite, or food/drug allergy. The swelling developed over a course of five to six hours. On inspection, the patient appeared to be in mild distress. His heart rate was 110 bpm, respiratory rate was 25 breaths/min, oxygen saturation was 98% on room air, and blood pressure was within the normal range. His body temperature was 99°F. Mild swelling was evident around the eyes and lips. Multiple erythematous lesions consistent with urticaria were seen on the trunk and back. Throat examination failed to reveal any significant swelling of the uvula or epiglottis. Normal vesicular breath sounds were heard upon performing a thorough chest examination, and no evidence of stridor or wheezing was found. He was treated with antihistamines only. A number of urgent investigations including complete blood count (CBC), erythrocyte sedimentation rate/C-reactive protein (ESR/CRP), D-dimers, renal function tests (RFTs), and urinalysis were advised. He was kept under observation for a period of eight hours. His lab reports were unremarkable. The patient was allowed to proceed home after his swelling and urticaria had completely settled. He was prescribed an antihistamine, and the parents were instructed to report back immediately in case he developed any swelling or difficulty in breathing. They were instructed to follow up after one week for a repeat SARS-CoV-2 PCR test. The PCR test turned out to be negative, and thereafter the patient remained asymptomatic on further follow-up. Angioedema in this patient could not be associated with any particular etiology; therefore, COVID-19 infection was considered a probable cause.

## Discussion

It has been reported that SARS-CoV-2 may influence the activation of the complement system and perhaps cause degranulation of mast cells [3]. This may lead to dermatological manifestations seen in the form of urticaria and/or angioedema. The occurrence of angioedema alongside urticaria in SARS-CoV-2 infection in the pediatric population has rarely been reported. Similar to the phenomenon described above, our case presented with urticaria followed by the development of angioedema around the lips and eyes [4].

Angioedema is the swelling of the skin and underlying structures, which usually persists for several hours to a couple of days depending on severity. The release of substances, such as histamine and bradykinin, increases the capillary permeability, causing sudden extravasation of intravascular fluid into the interstitium, leading to the development of swelling [5]. Regions mostly affected are the face, feet, hands, and genitals, with the lips and area surrounding eyes (periorbital region) being the most prominent among all [6]. Urticaria may arise alongside angioedema where numerous elevated erythematous skin lesions occur forming a coalescence. Acute urticaria is frequently observed in the pediatric population and can be associated with a wide variety of viral infections, medicines, or food products. Hives accompanying angioedema must never be dismissed as benign, self-resolving symptoms as they could deteriorate in a relatively short time and lead to full-blown anaphylaxis [7].

Assessment of urticaria and angioedema involves systemic review and laboratory tests such as CBC and skin patch test to rule out or augment the findings revealed by the physical examination. However, evidence suggests that patient history may, in fact, be the single most important factor to be considered [8]. After extracting a detailed patient history, we were able to narrow down the possible etiologies that may have led to the presenting symptoms.

The infection caused by the SARS-CoV-2 virus has been extensively studied in children. A case series on COVID-19 patients involved more than 2,100 children. Although there were no specific details with regard to angioedema, it was revealed that almost 94% of the children had mild-to-moderate disease, a number of them being completely asymptomatic [9]. Treatment of COVID-19 symptoms in the pediatric population is less complicated than adults and is associated with a better prognosis. We observed an immediate regression of angioedema after treatment was administered to our patient. The child’s symptoms resided over the course of few hours without the need for hospitalization or aggressive management.

## Conclusions

COVID-19 is an infectious disease that has recently been linked to a number of dermatological findings. Infection due to coronavirus has generally resulted in a mild course of disease in children compared to adults. The findings of urticaria followed by angioedema in our patient were no less than rare manifestations of SARS-CoV-2. Symptoms occurring in a similar fashion should prompt physicians to consider an underlying COVID-19 infection as it is linked to an increasing number of variable presentations. Due to limited literature, more research will contribute to the knowledge database of physicians allowing the healthcare community to improve patient management significantly.
